# Environmental impact potential of insect production chains for food and feed in Europe

**DOI:** 10.1093/af/vfad033

**Published:** 2023-08-14

**Authors:** Sergiy Smetana, Anita Bhatia, Uday Batta, Nisrine Mouhrim, Alberto Tonda

**Affiliations:** DIL German Institute of Food Technologies e.V., Quakenbrück, Germany; DIL German Institute of Food Technologies e.V., Quakenbrück, Germany; DIL German Institute of Food Technologies e.V., Quakenbrück, Germany; UMR 518 MIA-PS, INRAE, Université Paris-Saclay, 91120 Palaiseau, France; UMR 518 MIA-PS, INRAE, Université Paris-Saclay, 91120 Palaiseau, France; Institut des Systèmes Complexes de Paris Île-de-France (ISC-PIF) – UAR 3611 CNRS, Paris, France

**Keywords:** environmental indicators, insect production chains, life cycle assessment, sustainability, sustainable food systems

**Implications**

- Insects can address sustainability issues associated with current food systems by providing an alternative protein source to address hunger and disease.
- Only the production systems that rely on side-stream heat and alternate energy sources may benefit from replacing compound feed production with insect value chains.
- Seventy-five percent to 93% of the effects of compound feed production on global warming potential, land use, and fossil resource shortages can be avoided.
- To fully assess the potential of insect production, it is critical to consider a wide range of sustainability indicators, including social, economic, and environmental aspects.

## Introduction

The current food systems are facing several sustainability problems. One major issue is the environmental impact of food production, which contributes to climate change, deforestation, and biodiversity loss (Poore and Nemecek, 2018). Additionally, current food systems depend on finite resources, such as water and fossil fuels, which are becoming increasingly scarce. Moreover, the current food systems have a significantly negative impact on human health, particularly the increasing incidence of chronic diseases linked to the overconsumption of animal products (Willett, 2013). Furthermore, the current food systems are inequitable, as they often fail to provide adequate access to food for marginalized communities and contribute to social and economic inequality (UN, 2019). As a result, it is crucial to develop sustainable food systems that can provide nutritious food for a growing population while minimizing negative environmental and social impacts.

Alternative proteins, such as plant-based, lab-grown meat and insects, can potentially address many of the sustainability issues associated with current food systems. For example, replacing animal-based products with plant-based alternatives has significantly reduced greenhouse gas emissions, land use, and water use associated with food production (Poore and Nemecek, 2018). Additionally, lab-grown meat has been proposed to reduce the environmental impact of meat production while still providing a high-quality source of protein (Post et al., 2020). Insects are also seen as a promising alternative protein source, they have a high nutritional value, are rich in protein, vitamins, and minerals, and have a lower environmental impact than traditional livestock (van Huis et al., 2013). Furthermore, alternative proteins can contribute to food security by reducing dependency on finite resources and increasing the resilience of food systems (Sexton, Garnett & Lorimer, 2019). Alternative proteins can also improve human health by providing nutritious food options and reducing the incidence of chronic diseases linked to the overconsumption of animal products (Willett, 2013)

While life cycle assessment (LCA) studies and other specific information about insect production can provide valuable insights into the environmental and nutritional aspects of insect-based food systems, it is important to note that these studies do not provide a comprehensive understanding of the sustainability potential of insect production chains on a European level. Factors such as social acceptability, economic feasibility, and production scalability are also crucial to consider when evaluating the sustainability of insect production chains (Gjerris, Gamborg & Röcklinsberg, 2016; Veldkamp et al., 2022). Additionally, the results of LCA studies on insect production can be affected by several variables, such as insect species, the type of feed used, and the production method, which can lead to varying results (Smetana et al., 2021). Therefore, it is important to consider a wide range of sustainability indicators, including social, economic, and environmental aspects, to fully evaluate the potential of insect production as a sustainable food system on the European level.

The aim of this study was to define the potential of insect production to improve the sustainability of the food system on the European level using a comprehensive assessment approach. This study relied on the FAO Sustainability Assessment of Food and Agriculture Systems (SAFA) guidelines to analyze published data on environmental indicators such as greenhouse gas emissions, land use, water use, biodiversity, energy, and animal welfare (FAO, 2014). SAFA is a comprehensive worldwide framework that evaluates sustainability across food and agriculture value chains. It serves as a universal benchmark for analyzing the interplay between various sustainability dimensions and identifying conflicts and opportunities for mutual benefits. By assessing these indicators, this study provides a holistic basis for the identification of the potential of insect production to tackle environmental hotspots of sustainable food systems on the European level.

SAFA concentrates on supply chains and enterprise(s) as elements of those chains. The LCA approach focuses on the evaluation of the environmental impacts of a product through its lifecycle, and, therefore, is not always suitable for the sustainability analysis of regions and countries. Similarly to LCA, SAFA covers multiple components of inputs, outputs, and environmental impacts; however, its focus on a larger system scale (value chains) enables a more comprehensive consideration of the scope of good governance and social well-being of sustainability (SAFA).

The current study concentrated only on the aspects of environmental integrity, including the quality of the atmosphere (greenhouse gas emissions), water, land, biodiversity, materials and energy, and animal welfare. The impact categories that have been selected are extensively used and established due to their rigorous research and inclusion in the most scientifically validated methodologies. By using these categories, the study can facilitate evidence-based decision-making towards sustainability and provide a more comprehensive assessment of environmental and social impacts.

## Atmosphere

According to the SAFA guidelines, the food system should be analyzed from a few aspects of the environment. From the impacts on the condition of the *atmosphere*, we relied on the accounting of greenhouse gas (GHG) emissions. In some initial studies (van Huis et al. 2013), it is indicated that the GHG emissions per kilogram of insect protein were lower than those for beef and pork but higher than those for chicken and fish. Similarly, a study by van Loon et al. (2018) found that the GHG emissions per kilogram of mealworm protein were lower than those for beef and pork but higher than those for chicken and fish.

Impacts associated with GHG emissions in insect production systems depend heavily on the use of diet. Thus, using a standard diet based on commercial or proprietary feed is associated with 2.3–3.1 kg CO_2_eq per kg of fresh insects produced (Oonincx and de Boer, 2012; Halloran et al., 2017). This aligns with the results found for 1 kg of dried larvae, which is 5.76 kg CO_2_eq (Bava et al., 2019), and for 1 kg of protein, which is 3.9–7 kg CO_2_eq (Halloran et al., 2017; Bosch et al., 2019). However, some studies have reported a higher carbon footprint of up to 21.1 kg CO_2_eq per kg of fresh larvae (Suckling et al., 2020) or 15–29 kg CO_2_eq per kg of protein (Ulmer et al., 2020) when the production systems are specific and so on not optimized for the production. These higher impacts can be attributed to the inclusion of frass application to the field as an emission factor (Suckling et al., 2020) or the analysis of a different production system with low technology readiness level (Ulmer et al., 2020).

The impacts associated with GHG emissions of insect production based on food processing by-products (food waste) can vary widely, from positively impacting the environment at −6.42 to 5.3 kg CO_2_eq for all functional units (Thévenot et al., 2018; Bosch et al., 2019; Smetana et al., 2019; Ites et al., 2020). The application of manure as feed for insects has a great potential for environmental improvement, but reviewed studies have indicated considerable environmental impacts from 0.77–12 kg CO_2_ eq per 1 kg of dried insects (Roffeis et al., 2017) to 1–7 kg CO_2_eq per 1 kg of proteins (Bosch et al., 2019).

In order to consider the potential improvement in GHG emissions of the European food system, we relied on the following considerations:

- insect can potentially substitute different, or all meat produced in Europe;
- insects can potentially substitute compound protein feed produced in Europe;
- insects can potentially substitute other products on one-to-one basis on a wet basis (fresh insects to fresh meat);
- data on the amount of meat produced were acquired from EUROSTAT for 2021 (“EUROSTAT, 2021”, 2021);
- data on the amount of compound feed produced in Europe were acquired for 2021 from FEFAC (Feed and Food, 2021);
- data on the environmental impacts of meat and feed was acquired from the Agri-footprint database (van Paassen et al., 2019) and economic allocation methods using IMPACT2002+ (Jolliet et al., 2003).

For the estimation of GHG emission changes with insects replacing conventional products, we considered two options for their impact: minimal (0.3 kg CO_2_eq per 1 kg of insect biomass) and maximal (3 kg CO_2_eq per 1 kg of insect biomass). This range was defined as the most beneficial for different insect species, resulting from the analysis above. The beef impact was 35.0; pork: 6.95 and poultry 5.97 kg CO_2_eq per 1 kg of meat. 1 kg of compound feed was responsible for 1.34 kg CO_2_eq.

**Figure 1.**
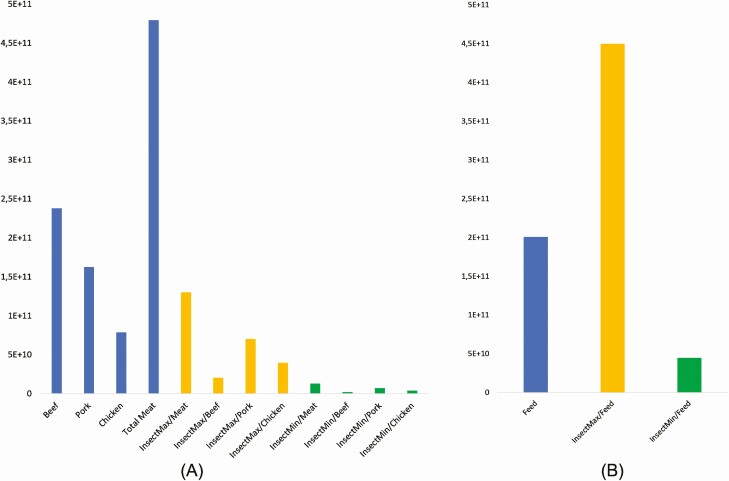
GHG emissions (in kg CO_2_ eq) associated with meat and feed produced in Europe and their changes due to potential substitution with insect biomass (A—meat production effects; B—compound feed production effects).

Production of insects with a defined range of GHG emission impact has the potential to improve the food system if the insects are consumed as a substitute for meats. GHG associated with meat can be reduced in this case by 72% to 97% (350–466 Mton CO_2_eq) (Figure 1). The biggest potential for impact reduction is observed in bovine meat production systems, the lowest in poultry substitution. The use of insects for feed substitution might not result in straightforward benefits, as a higher impact range would result in a higher environmental impact than the compound feed. A lower value could result in a 77% of impact reduction (155.9 Mton CO_2_eq). Substitution of meat in this case would have higher benefits (around 300 Mton CO_2_eq).

## Water footprint

Another key aspect of environmental influence is the *water footprint*. It is especially interesting as some studies indicate that the water use per kilogram of mealworm protein was lower than that for beef and pork, but higher than that for chicken and fish (van Loon et al. (2018); Huis et al. (2013)). It should be noted that water footprint is only indicated in a limited number of studies, with insects grown on a control diet resulting in 0.42–0.82 m^3^ of water depleted per 1 kg of fresh insects (Halloran et al., 2017; Suckling et al., 2020). The same impact is found for the protein-based unit at 0.71 m^3^ (Halloran et al., 2017). When calculated based on dry matter content, the impact increases to 1.26 m^3^ (Bava et al., 2019). The production of insects on by-products (food waste) results in varying levels of water depletion, from a low of 0.8–1.1 m^3^ per kg of dry matter content (Bava et al., 2019) to a high of 8.5–11 m^3^ per kg of fresh insects produced (Roffeis et al., 2017). The water footprint of insects produced on manure is also inconsistent, with ranges from a low of 8.5–11 m^3^ per 1 kg of insect on a dry matter basis (Roffeis et al., 2017) to a very high of 113.9–187.6 m^3^ (Roffeis et al., 2015). There is a lack of studies evaluating the water footprint of insects grown on food waste and manure.

Therefore, we further considered a range of potential water footprints of 0.4–0.8 m^3^ per 1 kg of insect biomass, which corresponds to the lower range of impacts of different insect species. Average beef was responsible for 0.25; pork for 0.05 and poultry for 0.067 m^3^ of water footprint per 1 kg of meat. Production of 1 kg of compound feed caused 0.0179 m^3^ of water footprint per 1 kg of feed.

**Figure 2.**
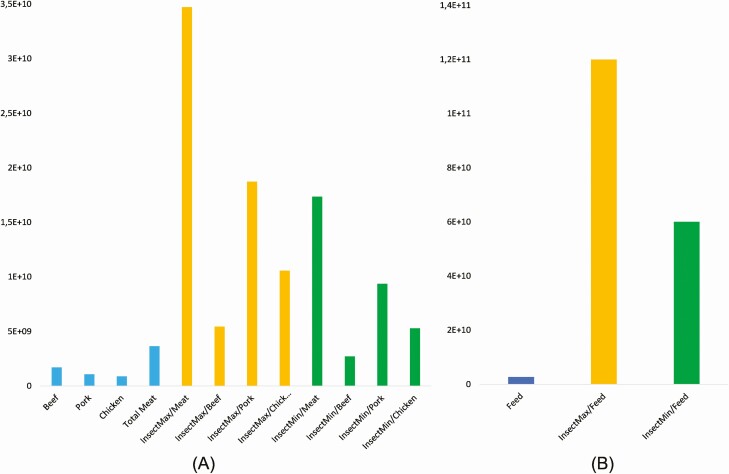
Water footprint (in m^3^) associated with meat and feed produced in Europe and their changes due to potential substitution with insect biomass (A—meat production effects; B—compound feed production effects).

Production of insects with a defined range of water footprint impacts is not expected to bring environmental benefits for Europe’s sustainable food systems (Figure 2). However, it should be noted that water footprint methods are often criticized for being under development and not reflecting the results with reliable certainty.

## Land use and biodiversity

Another aspect of sustainable food system assessment relates to the aspects of biodiversity change and changes in land use and soil quality. Despite the development of novel biodiversity assessment approaches, most LCA studies still rely on the *land use category* as a representation of the mentioned aspects. Land use of insect production indicated in studies in a wide range of impacts from 3.6 m^2^ per kg of fresh insects (Oonincx and de Boer, 2012) to as high as 94.7 m^2^ per 1 kg of insects on a dry matter basis (Bava et al., 2019) and 1.1–93 m^2^ per 1 kg of proteins (Bosch et al., 2019; Ulmer et al., 2020). Using by-products and wastes in the feed of the insects should lower the impacts to 1.6 m^2^ per 1 kg of fresh insects produced (Thévenot et al., 2018); -16.8 to 7.7 m^2^ per 1 kg of insect on a dry matter basis (Roffeis et al., 2015; Bava et al., 2019; Smetana et al., 2019; Ites et al., 2020) and 0–1 m^2^ per 1 kg proteins (Bosch et al., 2019).

The current study considered the land use impact of sustainable insect production in Europe in the range of 0.36–3.6 m^2^ per 1 kg of insect biomass. Such a range reflects the 10-fold range of lowest land use impacts indicated for insect production in studies. Average beef was responsible for 23.1; pork for 6.28, and poultry for 4.64 m^2^ of land per 1 kg of meat. It was necessary to use 1.48 m^2^ of land to produce 1 kg of compound feed.

**Figure 3.**
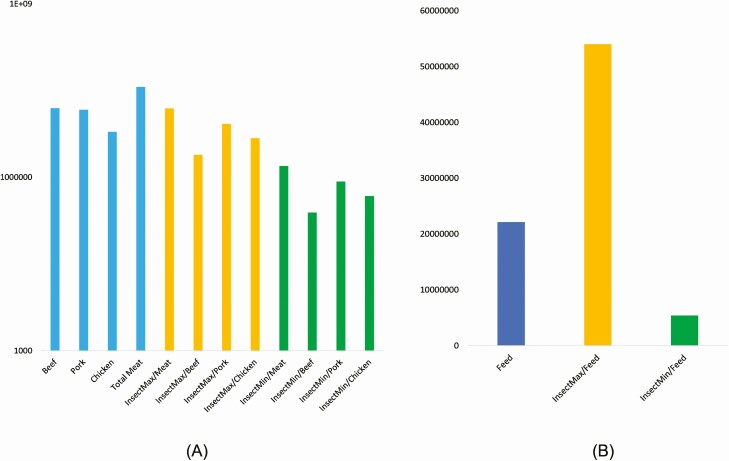
Land use (in ha) associated with meat and feed produced in Europe and their changes due to potential substitution with insect biomass (A—meat production effects (log scale); B—compound feed production effects).

Defined potential sustainable impacts associated with land use and biodiversity of insect mass production for food and feed in Europe allowed us to hypothesize about the potential to improve the food system if the insects are consumed as a substitute for meat and compound feed. Reduction of land use for meat production can be associated with substitution with insect biomass and reduction of land use in the scope of reduced in this case on 58% to 96% (209–350 Mm^2^). The biggest potential for impact reduction is observed in bovine meat production systems, the lowest in pork substitution (Figure 3). The use of insects for feed substitution might not result in straightforward benefits, as a higher impact range would result in higher environmental impacts than the compound feed. A lower value could result in a 75% impact reduction (167.5 Mm^2^). Substitution of meat can potentially result in higher environmental impact reduction (around 42 Mm^2^).

## Material and energy use

Material and *energy use* is an important factor, influencing the sustainability of food systems. Similar to previously mentioned factors, the energy use of insect production chains depends on the types of diets used (Smetana et al., 2021). Insect production on conventional diets results in energy use of 33.7 MJ per 1 kg of fresh insects (Oonincx and de Boer, 2012) and 159–425 MJ for 1 kg of proteins (Bosch et al., 2019 and Ulmer et al., 2020). Energy use for insect production, when grown on by-products and waste, is highly varied and ranges in the scope of -108 to 62.8 MJ per 1 kg of insect biomass on a dry matter basis (Roffeis et al., 2017; Thévenot et al., 2018; Ites et al., 2020) or 18–77 MJ per 1 kg protein (Bosch et al., 2019).

The current study relied on energy use impacts for insect production in Europe in the range of 0.36–21.2 MJ per 1 kg of insect biomass. The range is defined from the studies of the best insect production chains. Average beef was responsible for 104.0; pork 28.3 and poultry for 23.8 MJ of non renewable energy per 1 kg of meat. It was necessary 5.81 MJ of energy to produce 1 kg of compound feed.

**Figure 4.**
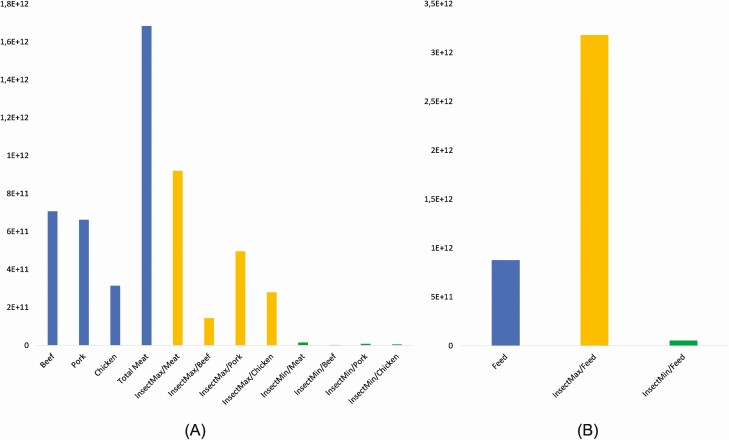
Non renewable energy use (in MJ) associated with meat and feed produced in Europe and their changes due to potential substitution with insect biomass (A—meat production effects; B—compound feed production effects).

The mass production of insects for food and feed in Europe has led us to consider their potential to reduce the dependency on non renewable energy sources. Reduction of the use of non renewable energy sources can be associated with the substitution of meat with insect biomass and can be expected in the range of 45% to 99% (763.5–1,668 PJ) (Figure 4). The biggest potential for impact reduction is observed in bovine meat production systems, the lowest in chicken substitution. However, in the case of energy-efficient insect production systems—the impact is minimal in all the scenarios. However, it is important to note that using insects for feed substitution may not necessarily result in straightforward benefits, as the environmental impacts could be higher than that of traditional compound feed. More energy-efficient insect production scenarios could result in a 93% of impact reduction (821.9 PJ). Therefore, the insect may be an energy-efficient substitute for both meat products and compound feeds.

## Animal welfare

One more important factor, with growing importance, relates to *animal welfare*. The animal welfare implications of insect production for food and feed in the European Union (EU) have not been extensively studied. However, a study by van Huis et al. (2013) suggests that compared to traditional livestock production, insect farming can provide a more humane environment for the insects, as they can be reared in smaller spaces, with less stress and better access to food. The humane environment is not the only ethical factor, and among others indicated for insects are environmental impact, human and animal health, human preferences and social acceptability, animal welfare, and broader animal ethics issues (Gjerris et al., 2016). The ethical issues related to animal integrity, death, and naturalness have been extensively studied in livestock farming and aquaculture for many years (van Huis (2019)). Established considerations and rules should be reevaluated in the context of insects and insect rearing, even though the concept of death and integrity, and even the phenomenological analysis of the possibility of experiencing empathy towards insects as a basis for including insects in the moral community, may seem unusual at first glance. Nevertheless, some authors point out that causing death is worthy of ethical consideration in and of itself, regardless of whether the animal suffered at the moment of killing. Taking the death considerations in the life cycle assessment perspective, some proposed approaches (Scherer et al., 2017) count for the number of animals needed to deliver the same function (e.g., amount of food). Obviously, such an approach discourages insect consumption for food or feed. Considering the number of animals killed, it could potentially be considered that using insects for food and feed could be very animal-unfriendly and unsustainable from this perspective (van Huis (2019)). However, in contrast, Fischer (2016) and van Huis (2019) consider that to produce plants for food of feed billions of insects have to be killed by insecticides or directly through harvesting thus making current food responsible for animal welfare issues associated with insect-killing in upstream processes, which are not being accounted currently.

While SAFA methodology allows for a comprehensive analysis and has the potential to include the trade-offs and complementarities between environmental, social, and economic aspects, it lacks the reflection of some key criteria important for the food systems. No indicators for assessing eutrophication, acidification or ecotoxicity are set in the SAFA framework (SAFA). Further analysis of such factors (which are currently still lacking in LCA studies) should improve the previous estimate presented in the current work. At the same time, this study concentrated on the assessment environmental integrity part of SAFA and did not target the aspects of good governance, economic resilience, and social well-being. The primary reason for the inclusion of environmental factors only relates to the limitations on the availability of studies covering aspects of, e.g., corporate ethics or holistic management, as well as fair trade practices or cultural diversity of evolving insect production industry. Such aspects are not well studied.

The current study, however, allows defining the feasible trends for industry development from aiming to reduce the environmental impact. Other factors might add another layer of trade-offs between different aspects and scenarios, but they would not change the defined positive potential of environmental improvement in a few impact categories due to the substitution of meat with insect-based products or lower potential for environmental impact improvement due to feed substitution.

## Conclusions

The study aims to define the potential of insect production for improving sustainable food systems on a European level utilising a comprehensive assessment approach. The study preferred that insects replete with a high nutritional value, are rich in protein, vitamins, and minerals and potentially have a lower environmental impact than traditional livestock, and thus can be an alternative protein source to address sustainability issues associated with current food systems. The study relied on modelling the change of crucial sustainability aspects defined in FAO Sustainability Assessment of Food and Agriculture Systems (SAFA) guidelines. It allowed determining that environmentally beneficial insect value chains can reduce the impact of livestock production systems by 40% to 97% in categories of global warming potential, land use, and fossil resources scarcity. It is possible if insect biomass substitutes meat (beef, pork, and poultry) efficiently produced. Substitution of compound feed production with insect value chains could be environmentally beneficial only in the cases of extremely efficient (lowest environmental impacts, e.g., insects grown on wastes or low-cost feeds, relying on side-stream heat and alternative energy sources) production systems. In this case, the 75% to 93% of impacts of compound feed production in categories of global warming potential, land use, and fossil resources scarcity can be eliminated. The study demonstrates that the insect industry in Europe should target two potential development scenarios, which result in positive environmental integrity results. The first scenario concentrates on the development of companies targeting the production of high-quality biomass for food (meat substitution approach). And the other one is for the companies targeting waste treatment and further use of insect biomass for feed purposes.

It is necessary to note that insect biomass did not demonstrate potential benefits in the water footprint and animal welfare impact categories. This is partially connected to the lack of assessments performed with the latest methodological developments or with a complete lack of specialised methods (e.g., animal welfare for insects). Additionally, the results of LCA studies on insect production can vary depending on the specific species of insect, type of feed used, and production method. Assessment of environmental integrity according to the SAFA framework does not necessarily include the impact categories of eutrophication, acidification and ecotoxicity. These aspects are traditionally important for food system value chains, and their assessment can provide valuable insight into trade-offs between different factors of environmental impact.

Moreover, it is essential to consider various factors, such as social acceptability, economic feasibility, and production scalability, when evaluating the sustainability of insect production chains. Moreover, the SAFA framework includes indicators of good governance, social resilience, and social well-being, which were not analyzed in this study. There is a need for holistic research studies that would use the SAFA framework to tackle insect production chains. Therefore, it is crucial to consider a wide range of sustainability indicators, including social, economic, and environmental aspects, to fully evaluate the insect’s production potential as a sustainable food system on the European level. The insect industry can explore these development scenarios and work towards establishing efficient insect production systems. Further research could be conducted to optimize insect rearing, processing, and utilization for various food and feed purposes. Efforts could also be made to address regulatory and consumer acceptance barriers that hinder the growth of the insect industry. The prospect for the insect industry appears promising as it offers a potential solution to the sustainability challenges associated with current food systems. As such, the industry can contribute to building a more resilient and sustainable food system for the future.

## About the Author

**Dr. Sergiy M. Smetana** works as a Head of Food Data Group at the German Institute of Food Technologies (DIL e.V.) since 2017. However, he joined the institute in 2013 and was responsible for sustainability assessment of food and food technologies. Before that he worked as a Visiting Fulbright Scholar in Brook Byers Institute of Sustainable Systems (Georgia Institute of Technology, Atlanta, GA, USA) for a year and six years as a Leading Engineer in Institute of Nature Management and Environmental Problems (Ukraine). The focus of his research is sustainability assessment of innovative food processing technologies, alternative protein sources and data analysis of complex food systems. Corresponding author: smsmetana@gmail.com

**Mrs. Anita Bhatia** is a Ph.D. student (Universität Osnabrück, Germany) on the topic “Data-driven Intelligent simulation model of global obesity”. Ph.D. is based on implementing complex system models using the white-box explainable machine learning models. She is a Research Associate at DIL, working within the Food Data Group since 2020, she is an experienced Senior Software Engineer with a demonstrated history of working in the internet and e-commerce industry. Skilled in Unit Testing, PHP, Python, jQuery, My SQL, CodeIgniter, and HTML. Strong engineering professional with a Master’s degree focused in Computer Science from NIT HAMIRPUR.

**Mr. Uday Kumar Batta** is an environmental engineer with expertise in sustainability and life cycle assessment (LCA) in the food and electrical industries. As an LCA expert, he has a deep understanding of LCA and its applications in sustainability and environmental management. His expertise in LCA allows him to provide valuable insights and recommendations to organizations on reducing their environmental footprint and moving toward more sustainable practices. As a graduate with a master’s degree in Environmental Engineering at SRH Hochschule Heidelberg, he has a strong foundation in environmental problems, guidelines, and technologies. His bachelor’s degree in electrical and electronics engineering allowed him to apply, design, and develop innovative solutions to real-world problems.

**Dr. Nisrine Mouhrim** is a postdoctoral fellow at the National Research Institute for Agriculture, Food and the Environment (INRAE) and member of the UMR MIA-Paris-Saclay, AgroParisTech. Her research areas of interest include multiobjective optimisation, metaheuristics, robust optimisation, and transportation issues.

**Dr. Alberto Tonda** received his Ph.D. in Computer Science Engineering from Politecnico di Torino (Italy) in 2011. After post doctoral experiences at the Complex Systems Institute of Paris and INRIA Saclay, he is currently a senior permanent researcher (DR) at the French National Institute of Research in Agriculture and Environment (INRAE). His research interests include semi supervised modeling of complex systems, evolutionary optimization, and machine learning, with main applications to food science and industry. Alberto Tonda authored more than 40 scientific papers published in refereed international journals, two books and over 70 contributions to international conferences. He led COST Action CA15118 FoodMC (2016-2020), focused on mathematical and computer-based models for food science and industry; he coorganized several workshops at the GECCO conference between 2016 and 2020; he is part of the editorial board of the journal Genetic Programming and Evolvable Machines (Springer), and editor for Engineering Applications of Artificial Intelligence (Elsevier).
